# Network Pharmacological Analysis and Animal Experimental Study on Osteoporosis Treatment with GuBen-ZengGu Granules

**DOI:** 10.1155/2023/9317557

**Published:** 2023-01-12

**Authors:** Kai Wang, Kai Fan, Hao-Nan Wen, Yun-Xiang Hai, Yan-Long Gong, Zhi-Jing Song, Wan-Tao Dong, Yi-Wei Jiang, Min Song

**Affiliations:** ^1^Clinical College of Chinese Medicine, Gansu University of Chinese Medicine, Lanzhou 730000, Gansu Province, China; ^2^Gansu Province Emergency Medical Aid Center, Lanzhou 730030, Gansu Province, China; ^3^Department of Orthopedics, The Affiliated Hospital of Gansu University of Chinese Medicine, Lanzhou 730020, Gansu Province, China

## Abstract

**Aim:**

We explored the molecular pathway and material basis of GuBen-ZengGu granules (GBZGG) in treating osteoporosis using network pharmacology and animal experiments.

**Methods:**

The effective active components and potential targets of GBZGG were obtained from the TCMSP database and BATMAN-TCM database. Disease-related genes were obtained from GeneCard, NCBI, and DisGeNET. Next, a protein interaction network was established using the STRING database, and core genes were screened using the MCODE module. Cytoscape 3.8.0 was used to construct the network of component-disease-pathway-target, and KEGG pathway enrichment analyses were performed using the clusterProfiler R package to predict the mechanism of GBZGG in treating osteoporosis. An osteoporosis rat model was established by ovarian excision (OVX), and the partial results of network pharmacology were experimentally verified.

**Results:**

Pharmacodynamic results showed that GBZGG increased bone mineral density (BMD) and significantly improved the indexes of femur microstructure in model rats. The network pharmacology results showed that quercetin, luteolin, stigmasterol, angelicin, kaempferol, bakuchiol, bakuchiol, 7-O-methylisomucronulatum, isorhamnetin, formononetin, and beta-sitosterol are the major components of GBZGG, with MAPK1, AKT1, JUN, HSP90AA1, RELA, MAPK14, ESR1, RXRA, FOS, MAPK8, NCOA1, MYC, and IL-6 as its core targets for treating osteoporosis. Biological effects could be exerted by regulating the signaling pathways of fluid shear stress and the signaling pathways of atherosclerosis, advanced glycation end products (AGE-RAGE) of diabetic complications, prostate cancer, interleukin (IL-17), tumor necrosis factor (TNF), hepatitis B, mitogen-activated protein kinase (MAPK), etc. The results of animal experiments showed that GBZGG could reduce the serum levels of IL-6 and TNF-*α*, increase the expression of bone morphogenetic protein-2 (BMP-2) and runt-related transcription factor 2 (RUNX2) protein, and inhibit the activity of extracellular-regulated protein kinases (ERK1/2) and phosphorylation ERK1/2 (p-ERK1/2) protein.

**Conclusion:**

GBZGG reduces the expression of ERK1/2 and p-ERK1/2 proteins and mRNAs through the inhibitory effects on IL-6 and TNF-*α* and negatively regulates the MAPK/ERK signaling pathway. The osteoporosis model showed that it effectively improved the loss of bone mass and destruction of bone microstructure in rats and maintained a positive balance for bone metabolism.

## 1. Introduction

Osteoporosis (OP) is a systemic metabolic bone disease characterized by disruption of bone microarchitecture and impaired bone strength, resulting in increased susceptibility to fractures [1]. With the increasing incidences of OP annually and changes in the global population structure, fragility fractures in the spine, extremities, and other components at a later stage, it has started seriously affecting the quality of life of patients and significantly increased the burden on health care. The current treatment for OP aims at reducing discomfort, delaying progression, reducing the risk of fracture, and improving the quality of life of patients.

Traditional Chinese medicine (TCM) follows the principles of “syndrome differentiation and treatment variation” to regulate the body as a whole and exerts the regulatory effects of multiple molecules, targets, and pathways in preventing and treating OP. This approach has been accepted and recognized by an increasing number of patients because of its advantages of simplicity, convenience, examination, and cheapness. TCM believes that “kidneys store the essence to generate the marrow,” and the health of bones depends on the sufficiency of kidney essence, whereas kidney deficiency and essence reduction largely contribute to OP [2]. In addition, it is intricately related to the decline of the digestive system, especially the functions of the spleen and stomach. Therefore, tonifying the kidneys and filling the essence are the roots of TCM in preventing and treating this disease, and tonifying the spleen, supplementing Qi, harmonizing the blood, and dredging collaterals are the core concepts of OP treatment [3]. GuBen-ZengGu granules (GBZGG), which are composed of nine kinds of Chinese herbal medicines, namely, Radix Astragali (Huangqi, HQ), Epimedii Folium (Yinyanghuo, YYH), Codonopsis pilosula (Dangshen, DS), Angelicae Sinensis Radix (Danggui, DG), Cistanches Herba (Roucongrong, RCR), Rehmannia glutinosa (Shu Di Huang, SDH), Psoraleae Fructus (Buguzhi, BGZ), Cibotii Rhizoma (Gouji, GJ), Linderae Radix (Wu Yao, WY), nine drugs in the compound prescription have been evaluated with http://www.worldfloraonline.org. GBZGG is a clinical compound preparation developed by Professor Min Song, a famous doctor of TCM in Gansu Province (GYZ20220417000). It has been used for more than 20 years. Clinical trials have demonstrated that it can increase bone mineral density (BMD), improve clinical symptoms, be safe and effective, have a few adverse reactions, and have an excellent therapeutic effect on OP [4]. Previous studies have reported that GBZGG-treated serum exerts bone marrow promoting, stromal cell proliferation, osteogenic differentiation, and inhibiting adipogenic differentiation effects, and its mechanism is intricately related to the regulation of key proteins in the Notch signaling pathway, Wnt/*β*-catenin signaling pathway, and bone morphogenetic protein (BMP) signaling pathway [5, 6]. GBZGG can bidirectionally regulate bone metabolism in a rat model of postmenopausal osteoporosis [7], exert estrogen-like effects to improve BMD in rats, and regulate intestinal bacterial disorders by activating the intestinal bacterial RNA translation and transcription and the generation and repair of the cell wall [8]. Although certain breakthroughs have been made at the cellular and molecular levels and proteomics, the mechanism of action of TCM compounds in treating OP has not been completely elucidated due to their diverse components and complex affinity targets. The interaction between molecules and different proteins results in a complex pharmacology of drugs. In this context, network pharmacology has emerged as a new disciplinary concept proposed in the context of big data [9] by constructing a drug-gene-target-disease network, combining the biological information with pharmacological analysis, and enriching the understanding of drug structural information and pharmacological effects from an interrelated perspective [10] based on the overall concept of TCM. Therefore, we used network pharmacology to analyze the active components, target genes, biological functions, and signaling pathways of GBZGG in treating OP and verified them through animal experiments. We believe this study will be highly significant in revealing the material basis and molecular mechanism underlying OP treatment and providing a scientific basis for the next step of research to broaden the ideas of TCM in preventing and treating OP.

## 2. Materials

### 2.1. Experimental Animals

Specific pathogen-free (SPF) grade SD female rats, aged 8 weeks and weighing about 180–200 g, were provided by the Animal Experimental Center of Gansu University of TCM. The experimental site was the SPF-grade animal laboratory of the Animal Experimental Center of the Gansu University of TCM. The room temperature was regulated at 22 to 26°C, and the air humidity varied from 45 to 65%. Six animals were housed in each cage; the light duration was 7:00 AM to 7:00 PM; sterile animal feeds were regularly and quantitatively fed; free access was provided to sterilized distilled water; and the bedding was changed every 5 days. The experimental procedures were conducted according to the ethical review requirements for animal experiments of the Gansu University of TCM and followed the 3R principle, document approval no. 2020–310.

### 2.2. Experimental Drugs

#### 2.2.1. Formula GBZGG

The drugs are Radix Astragali 40 g (batch number: 0103083), Epimedii Folium 15 g (batch number: 0100493), *Codonopsis pilosula* 12 g (batch number: 0103183), Angelicae Sinensis Radix 12 g (batch number: 0093243), Cistanches Herba 12 g (batch number: 0105493), *Rehmannia glutinosa* 12 g (batch number: 0101523), Psoraleae Fructus 12 g (batch number: 0096913), Cibotii Rhizoma 12 g (batch number: 0113383), and Linderae Radix (batch number: 0091233). Estradiol valerate (Bayer HealthCare, batch number: 650A) was provided by the Pharmaceutical Preparation Center of the Affiliated Hospital of the Gansu University of TCM.

### 2.3. Main Reagents

Tumor necrosis factor-*α* (TNF-*α*) and interleukin-6 (IL-6) kits were purchased from Jiangsu Masha Industrial Co., Ltd. (F3056-A and F3066-A, respectively). The main results are runt-related transcription factor 2 (Runx2), BMP-2, extracellular-regulated protein kinases (ERK1/2), and phosphorylation-induced ERK1/2 (p-ERK1/2) protein antibodies (Bioss, bs-1134R, bs-1012R, bs-0022R, bs-3292R), ERK1 + p-ERK1 protein antibody, and ERK2 + p-ERK2 protein antibody (Abcam, ab19282, ab32081).

### 2.4. Instruments and Equipment

Dual-energy X-ray BMD instrument (GE, USA), viva CT80 Micro-CT scanner (SCANCO, Switzerland), microplate reader (Bio-Rad, USA), electrophoresis apparatus, diaphragm instrument (Beijing Liuyi Biotechnology), vortex oscillator, Minifuge (SeloCzech, USA), and NanoDrop nucleic acid concentration meter, real-time quantitative PCR instrument (Thermo Fisher Scientific, USA) were used.

## 3. Methods

### 3.1. Pharmacodynamic Studies

#### 3.1.1. Animal Grouping, Modeling, and Administration

The rats were randomly divided into the sham group, ovarian excision group (OVX group), estradiol valerate group (EV group), GBZGG high-dose group (GBHD group), GBZGG medium-dose group (GBMD group), and GBZGG low-dose group (GBLD group), with 10 animals in each group. In the sham group, adipose tissue was harvested at approximately similar positions after skin incision, whereas in the other group, OVX was performed, and successful modeling was judged by BMD testing. GBZGG was converted into rat equivalent doses according to the adult dose (body weight of 70 kg) and divided into different concentrations of 24.4 g·kg^−1^, 12.2 g·kg^−1^, and 6.1 g·kg^−1^ corresponding to the GBHD group, GBMD group, and GBLD group, respectively. In the EV group, rats were intragastrically administered estradiol valerate tablets administered at a concentration of 0.09 mg·kg^−1^. The sham group and OVX group received an equal volume of normal saline. Different concentrations of the drug were prepared as 2 mL of the solution according to the rat gavage volume of 10 mL·kg^−1^ standard for gavage once daily for 12 weeks. To ensure stable intragastric drug concentration and reduce concentration error, intragastric rats were weighed and measured regularly every week, and the intragastric concentration was adjusted according to the changes in their body weights.

#### 3.1.2. Bone Mineral Density Detection

After the completion of drug intervention in each group of rats, anesthesia was induced by intraperitoneally injecting 1% pentobarbital sodium 3 mL·kg^−1^. The rats were placed in the prone position on the operating table for dual-energy X-ray BMD meter measurement of the total body BMD. Selection of the measurement type is as follows: the whole body of the small animal, standard mode, measurement parameters, length: 75.7 cm, width: 25.0 cm, exposure parameters, voltage: 76 kV, current: 0.15 mA, exposure time: 4:08 (min: sec), and scanning dose: 1.8 *μ*Gy. BMD unit: g/cm^2^.

#### 3.1.3. HE Staining of Bone Tissue

The ipsilateral femurs of rats in each group were sampled and fixed using 4% paraformaldehyde for 24 h before being transferred into the EDTA decalcification solution for decalcification treatment. The decalcification solution was replaced once a week for 8 weeks. After decalcification, specimens were trimmed to an appropriate size for paraffin embedding, and the embedded paraffin blocks were cut into 5 *μ*m thick slices on a microtome, flattened in hot water, and transferred to glass slides. Finally, the slices were deparaffinized and stained, and histopathological changes in the femur were observed under an electron microscope.

#### 3.1.4. Micro-CT Detection Methods

To perform micro-CT examination, femur samples were soaked in the paraformaldehyde fixative. The distal end plate of the femur was selected as the starting scanning site and scanned upward successively (parameter settings: scanning energy/intensity: 55 kV, 145 *μ*A, 8 W, slice distance: 10.4 *μ*m, image matrix: 3078 × 3072 × 336 pixels, integration time: 350 ms, rotation angle: 0°, number of slices: 336). After scanning, a two-dimensional (2D) image of the region was created. The micro-CT scanner's built-in drawing software was used to select the cancellous component as the region of interest (ROI) for 3D figure drawing (number of consecutively drawn layers: 200 slices). The values of bone morphology-related parameters were operationally analyzed for statistical analysis.

### 3.2. Network Pharmacology Analysis

#### 3.2.1. Query of Target Information of Active Ingredients of GBZGG

Oral drugs are primarily absorbed through small intestinal epithelial cells, and their oral bioavailability (OB) is affected by small intestinal epithelial cell drug permeability (Caco-2) proteins. Whether drug lead compounds can be drug-like (DL) is evaluated. Seven pharmaceutical compound components, namely, Radix Astragali, Angelicae Sinensis Radix, *Codonopsis pilosula*, Epimedii Folium, *Rehmannia glutinosa*, Cistanches Herba, and Linderae Radix, were retrieved from the TCMSP database (http://tcmspw.com/tcmsp.php) using parameters “OB ≥ 30% and DL ≥ 0.18” as screening conditions. Cibotii Rhizoma and Psoraleae Fructus were not included in the TCMSP database; the compound components of the two were supplemented from the Batman-TCM database (http://bionet.ncpsb.org.cn/batman-tcm/), and the component targets with scores >20 were selected for inclusion. All targets were corrected by the UniProt database (https://www.uniprot.org/) to remove nonhuman targets.

#### 3.2.2. Acquisition of Disease Targets

“Osteoporosis” was entered as a keyword into the GeneCards database (https://www.genecards.org/), NCBI gene database (https://www.ncbi.nlm.nih.gov/), and DisGeNET database (https://www.disgenet.org/) for human gene search to select OP-related disease targets.

#### 3.2.3. Construction of the GBZGG-Component-Target-OP Network

The VennDiagram R software (https://cran.r-project.org/web/packages/VennDiagram/) was used to construct the intersection target genes of the active ingredients of the obtained GBZGG and the OP disease target. A Venn diagram was drawn. To better understand the complex action relationship between compound components, diseases, and corresponding targets, the Cytoscape 3.8.0 software (https://cytoscape.org/) was used to construct a GBZGG-component-target-OP network diagram and calculate the corresponding relationship nodes.

#### 3.2.4. Protein Interaction Network and Cluster Analysis

The common targets of GBZGG and OP were entered into the STRING database (https://www.string-db.org/), and protein-protein interaction (PPI) network was constructed. The biological species was set as “*Homo sapiens*.” The disconnected nodes were eliminated using a confidence >0.9 as a screening condition to obtain the PPI network. The PPI network was imported into Cytoscape 3.8.0, and the MCODE module was used to analyze gene clusters as well as screen core targets.

#### 3.2.5. Enrichment Analysis of Common Target Genes

The KEGG enrichment analyses of target genes of GBZGG in treating OP were performed using the clusterProfiler R software package (*p* value cut-off <0.05, *q* value cut-off <0.05) to predict the related signaling pathways involved in the regulation of core genes in the treatment of OP by GBZGG. Finally, the “component-disease-signalingpathway-target” network diagram was constructed using Cytoscape 3.8.0.

### 3.3. Animal Experimental Studies

#### 3.3.1. Animal Grouping, Modeling, and Administration

It is the same as described in Section “3.1.1.”

#### 3.3.2. Serum IL-6 and TNF-*α* Content Evaluation by ELISA

After the rats were anesthetized, blood sampling from the heart was performed using the “three-line localization” method. The collected blood was allowed to stand at room temperature for 2 h. It was centrifuged at 3000 rpm at room temperature for 15 min. The separated upper serum was aspirated using a micropipette. The levels of IL-6 and TNF-*α* in the serum were determined using the ELISA kit, and the microplate was read at 450 nm.

#### 3.3.3. Detection of BMP-2, Runx2, and ERK1/2 mRNAs by RT-PCR

The femoral samples were frozen at –80°C and cut into pieces. Next, 100 mg of samples from each group were placed into a mortar for grinding. They were pulverized into powder form and transferred into a centrifuge tube. Afterward, 1 mL of RNAiso Plus was added to dissociate the ribosomes. Next, 0.2 mL of chloroform was added and allowed to stand at room temperature for 3 min. It was centrifuged at 12,000 rpm for 10 min at 4°C, and the upper aqueous phase was drawn into a new centrifuge tube, to which 0.5 mL of isopropanol was added and which was made to stand at room temperature for 10 min. It was again centrifuged at 12,000 rpm for 10 min at 4°C and 1 mL of 75% ethanol was added, washed thoroughly by vortexing, and centrifuged at 7500 rpm for 5 min at 4°C. The RNA was dissolved by adding 50 *μ*L of enzyme-free water, and the RNA loading concentration was calculated. It was reverse transcribed into cDNA according to the instructions provided in the reverse transcription kit. The results were calculated by the 2^–△△CT^ method, and the data were analyzed by statistical software to analyze the mRNA content of each group of samples. The primer sequences used are shown in Table 1.

#### 3.3.4. BMP-2, ERK1/2, p-ERK1/2, and Runx2 Protein Expression by Western Blotting

Frozen femoral samples were cut into pieces and placed in 2 mL centrifuge tubes and ground with a high-speed and low-temperature tissue grinder. A total of 100 mg of sample was transferred to a 2 mL centrifuge tube, and 1 mL of RIPA lysate containing PMSF was added. It was completely lysed on ice and centrifuged at 12,000 rpm for 15 min at 4°C; the supernatant was aspirated, and a BCA assay was used for protein quantification. The protein was mixed with the sample buffer in a ratio of 4 : 1 and denatured with boiling water for 5 min. Electrophoretic separation was performed at a protein loading volume of 30 *μ*L, transferred to polyvinylidene fluoride (PVDF) membranes, and blocked with 5% non-fat dry milk for 90 min. They were placed in protein primary antibody (BMP-2, Runx2, EKR1/2, p-ERK1/2) dilutions (1 : 1000) and incubated overnight at 4°C on a shaker, followed by incubation with II antibody for 1 h. The luminescent solution was mixed in equal proportion, shaken well, and added to the PVDF membrane. The membrane was developed and observed after exposure, and the bands were quantitatively analyzed using the ImageJ software.

#### 3.3.5. Immunohistochemistry for ERK1/2 and p-ERK1/2 Protein Expression

The prepared paraffin sections were deparaffinized and hydrated with xylene and ethanol. The citric acid antigen was heat-repaired, washed with phosphate-buffered saline (PBS), and subsequently incubated with 3% H_2_O_2_ for 15 min at room temperature. The sections were blocked with sera homologous to the secondary antibody and incubated at 37°C for 30 min. Primary antibodies (ERK1/2 and p-ERK1/2 antibodies) were added dropwise and incubated overnight in a refrigerator at 4°C. Sections were removed for recovery, washed, and dropped with secondary antibody, incubated at 37°C for 30 min, developed by adding 3, 3-diaminobenzidine (DAB), and stained for imaging. The Image Pro Plus software was used for data analysis.

#### 3.3.6. Statistical Analysis

The experimental data were statistically analyzed using SPSS 23.0 and GraphPad Prism 7 software. Measurement data are expressed as mean ± standard deviation (mean ± SD). An independent sample *t*-test was used for comparison between the two groups. One-way analysis of variance (ANOVA) was used for comparison between multiple groups. Statistical differences were accepted when the *p* value was <0.05.

## 4. Results

### 4.1. Pharmacodynamic Study Results

#### 4.1.1. BMD Test Results

**Figure 1:**
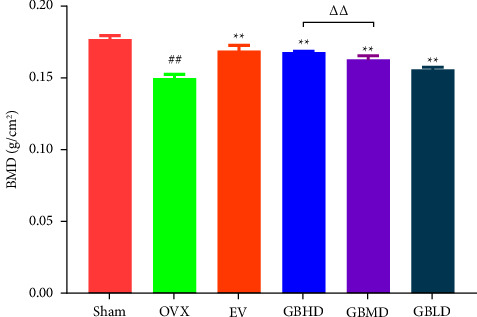
Changes of BMD in rats. ^##^*P* < 0.01 vs. the sham group; ^*∗∗*^*P* < 0.01 vs. the OVX group; ^ΔΔ^*P* < 0.01vs. the GBHD group. Mean ± SD, *n* = 3.

Compared with the sham group, the BMD of the OVX group was significantly lower (*P* < 0.01). Compared with the OVX group, the BMD of the EV, GBHD, GBMD, and GBLD groups was significantly increased (*P* < 0.01). The BMD of the GBHD group was significantly higher than that of the GBLD group (*P* < 0.01), as shown in Figure 1.

#### 4.1.2. Histopathological Observation of Bones

**Figure 2:**
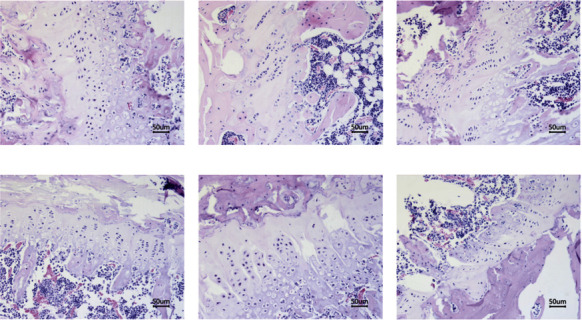
Pathological results of rat femur (HE staining, ×200). (a) Sham group; (b) OVX group; (c) EV group; (d) GBHD group; (e) GBMD group; (f) GBLD group. *n* = 3. Scale bar = 50 *μ*m.

The structure of the femoral bone was normal in the sham group, and the thickness and continuity of the bone under the epiphyseal plate were good. The structure was normal, and the number was higher, as shown in Figure 2(a). In contrast, the bone structure became thinner and sparsely arranged in the OVX group; the continuity was interrupted, the structure was incomplete, numerous blind ends appeared, the thickness of the small beam wall was unequal, and osteoblast and osteoclast proliferation was observed around the bone, as shown in Figure 2(b). Compared with the OVX group, the EV group had increased bone thickness, an elevated number of osteocytes, a gradually intact structure, and enhanced connection between the two ends of the bone, as shown in Figure 2(c). In the GBZGG groups, the bone structure gradually became intact with an increase in drug dose; the thickness increased, the number of osteocytes increased, the connection between the bone and the surrounding tissues increased, and the overall structure gradually became intact, as shown in Figures 2(d)–2(f).

#### 4.1.3. Micro-CT Bone Morphological Findings

**Figure 3:**
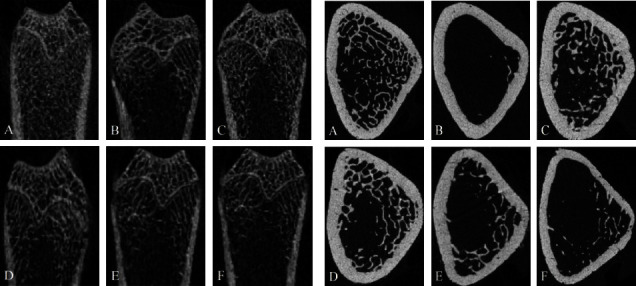
2D longitudinal and transverse scanning images of rat femur. (a) 2D longitudinal scanning images of rat femur; (b) 2D transverse scanning images of rat femur. (A) Sham group; (B) OVX group; (c) EV group; (D) GBHD group; (E) GBMD group; (F) GBLD group.

**Figure 4:**
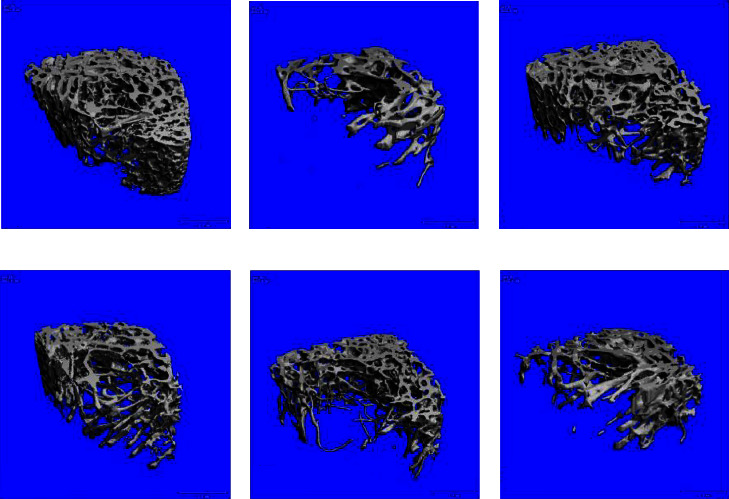
3D reconstructed image of the cancellous bone in the rat femur. (a) Sham group; (b) OVX group; (c) EV group; (d) GBHD group; (e) GBMD group; (f) GBLD group. *n* = 3. Scale bar = 1.0 mm.

Compared with the sham group, the OVX group showed significantly reduced the cancellous bone in the femoral cavity, sparse separation of the bone structure, and fracture of the connection, as shown in Figure 3. After three-dimensional reconstruction, the overall structure of the sham group degenerated, and the volume decreased. Following drug intervention, the number of bones increased significantly, the loose structure was improved, and the overall structure of cancellous bone was more complete. In addition, the effect was more evident in GBZGG groups with an increase in the dose, and the effect of GBHD was equivalent to that of EV, as shown in Figure 4.

#### 4.1.4. Changes in Femoral Bone Morphometric Parameters

Compared with the sham group, trabecular number (Tb.N) in the OVX group was significantly lower (*P* < 0.01). Compared with the OVX group, Tb.N number was significantly increased in the EV, GBHD, GBMD, and GBLD groups (*P* < 0.01). Compared with the sham group, the OVX group had a higher prevalence of trabecular thickness (Tb.Th), which was significantly decreased (*P* < 0.01). Compared with the OVX group, the Tb.Th of the EV, GBHD, GBMD, and GBLD groups was significantly improved (*P* < 0.05). Compared with the sham group, the trabecular separation (Tb.Sp) in the OVX group was significantly increased (*P* < 0.01). Compared with the OVX group, the Tb.Sp of the EV, GBHD, GBMD, and GBLD groups was significantly reduced (*P* < 0.01), as shown in Table 2.

Compared with the sham group, the OVX group connectivity density (Conn.D) was significantly reduced (*P* < 0.01). Compared with the OVX group, the Conn.D of EV, GBHD, GBMD, and GBLD groups was significantly increased (*P* < 0.01). Compared with the sham group, the Structure Model Index (SMI) was significantly higher in the OVX group (*P* < 0.01), whereas it was significantly decreased in the EV, GBHD, and GBMD groups (*P* < 0.01). Compared with the sham group, bone volume to tissue volume (BV/TV) was significantly decreased in the OVX group (*P* < 0.01), and compared with the OVX group, the bone volume fraction was significantly improved in the EV, GBHD, GBMD, and GBLD groups (*P* < 0.01), as shown in Table 3.

### 4.2. Network Pharmacology Analysis Results

#### 4.2.1. Prediction of Common Target Genes of GBZGG-OP

**Figure 5:**
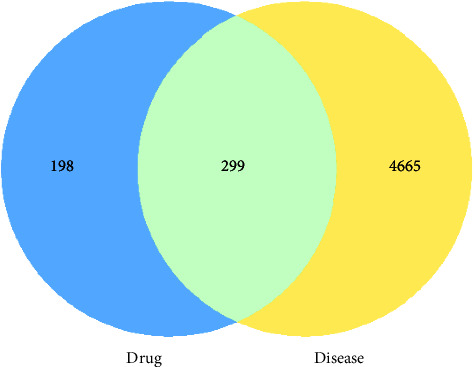
Venn diagram of common target genes between GuBen-ZengGu granules and OP. Note: blue represents the number of targets of active components of the drug, and yellow represents the number of disease-related genes, which are 299 cross-targeted genes.

After retrieval, Radix Astragali was predicted to contain 20 compound components and 194 targets, Angelicae Sinensis Radix had 2 compound components and 51 targets, *Codonopsis pilosula* had 21 compound components and 106 targets, *Rehmannia glutinosa* had 2 compound components and 30 targets, Epimedii Folium had 23 compound components and 208 targets, Cistanches Herba had 7 compound components and 159 targets, Linderae Radix had 9 compound components and 176 targets, Cibotii Rhizoma had 10 compound components and 50 targets, and Psoraleae Fructus had 14 compound components and 274 targets, as shown in Supplementary 1. We obtained 86 components of GBZGG compounds and 497 potential targets of action after summarizing and deweighting. In addition, 4,680 relevant targets were obtained from the GeneCards database, 459 relevant targets were obtained from the NCBI database, and 1,098 relevant targets were obtained from the DisGeNET database. Finally, 4,964 OP-related genes were obtained after merging and deleting the genes from these three databases, as shown in Supplementary 2. The selected drug targets and disease targets were input into the Venny 2.1 software to obtain 299 common targets, as shown in Supplementary 3. The common targets were subsequently used as predicted targets of drugs acting on diseases for the following pathway enrichment analysis, as shown in Figure 5.

#### 4.2.2. Construction of GBZGG-Ingredient-Target-OP Network

**Figure 6:**
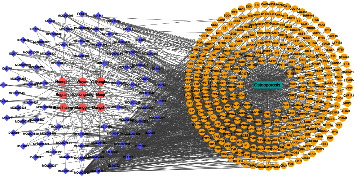
The GBZGG-ingredient-target-OP interaction network. Note: in the network, purple represents the active ingredient, yellow represents the target of the drug acting on the disease, pink represents the drug composed of GuBen-ZengGu granules, and the green rectangle represents osteoporosis.

The 299 common target genes obtained and the 86 effective active components of GBZGG were visualized using Cytoscape 3.8.0 software to construct a GBZGG-component-target-OP regulatory network, as shown in Supplementary 4. In all, 1,347 pairs of relationship data and 395 association nodes were collated, as shown in Figure 6. The GBZGG-ingredient-target-OP network was imported into Cytoscape 3.8.0 for topological analysis, and the components were ranked in degree. A higher value of components was related to a higher significance. The top 10 key components screened according to degree were quercetin, luteolin, stigmasterol, angelicin, kaempferol, bakuchiol, 7-O-methylisomucronulatum, isorhamnetin, and formononetin, as shown in Supplementary 5.

#### 4.2.3. PPI Network Construction Results

**Figure 7:**
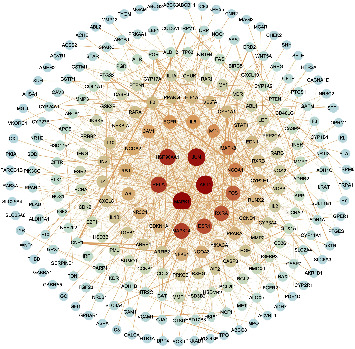
PPI network constructed by Cytoscape. Note: node color and size are adjusted according to the degree, the intensity of the red color is associated with the degree, and thickness of the line, from thickness to fine, indicates the edge betweenness from large to small.

**Figure 8:**
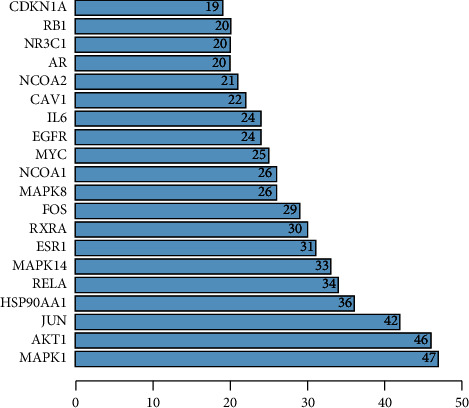
Bar graph of the number of adjacency nodes in the protein interaction network. Note: topological analysis was performed using the NetworkAnalyzer tool. Degree ranking showed that genes with scores greater than the average score were selected as key targets. A total of 87 key targets were selected. The top 20 targets were imaged using R 4.0.3, and the abscissa was the degree value of each target.

Among the PPI networks constructed using the STRING database, there are 299 nodes with 1,011 edges, and the average degree is 6.76, as shown in Figure 7. The results were imported into the Cytoscape 3.8.0 software to draw the PPI network and more intuitively display the protein interaction relationship, as shown in Figure 8. We used the NetworkAnalyzer tool to conduct the topological analysis, and by ranking, genes with scores greater than the average score were selected as key targets, as shown in Supplementary 6. A total of 87 key targets were screened, and the degree arrangement revealed MAPK1, AKT1, JUN, HSP90AA1, RELA, MAPK14, ESR1, RXRA, FOS, MAPK8, NCOA1, MYC, and IL-6 constituting the core position of the network.

#### 4.2.4. Cluster Analysis Results

The MCODE module analysis revealed 14 gene clusters and 13 core genes. *ADH1C*, *ESR1*, *NOS3*, *NCOA3*, *IL6*, *PCNA*, *CYP2R1*, *F7*, *NFE2L2*, *IRF1*, *VCAM1*, *MMP1*, and *TRPM8* belonged to the core genes, as shown in Table 4 and Supplementary 7.

#### 4.2.5. KEGG Pathway Enrichment Analysis Results

**Figure 9:**
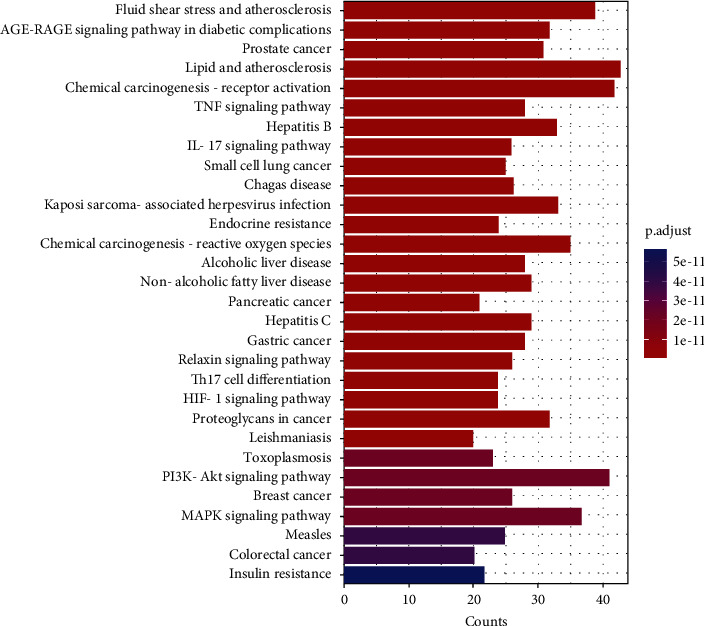
Histogram of KEGG enrichment analysis. Note: abscissa represents the number of genes enriched in KEGG, and the ordinate is the enriched KEGG Signal pathways. Color represents the statistical significance of enrichment, and high intensity of the red color indicates the degree of enrichment.

The KEGG pathway enrichment analysis revealed 184 signaling pathways involved in treating OP with GBZGG, majorly playing a role in treating OP by regulating the fluid shear stress and AGE-RAGE, prostate cancer, IL-17, TNF, hepatitis B, PI3K-AKt, MAPK, and other signaling pathways of atherosclerosis and diabetic complications, as shown in Figure 9 and Supplementary 8.

**Figure 10:**
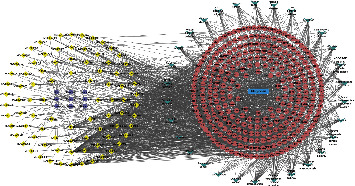
The component-disease-pathway-target interaction network. Note: the yellow color in the figure represents the compound, the pink color represents the targets of the disease, the green color represents the most significant top 30 signaling pathways, the blue color represents osteoporosis, and purple color represents the drug composed of GuBen-ZengGu granules.

To more visually display the characteristics of multicomponent and multi-target effects of active ingredients of TCM compounds in treating diseases, we created a pathway network diagram using the Cytoscape3.8.0 software, as shown in Figure 10 and Supplementary 9.

### 4.3. Results Verified by Animal Experiments

#### 4.3.1. Serum IL-6 and TNF-*α* Expression

**Figure 11:**
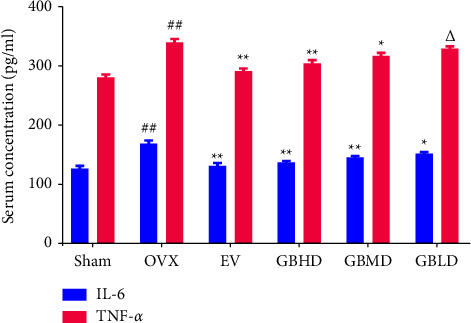
Changes in serum IL-6 and TNF-*α* levels. ^##^*P* < 0.01 vs. the sham group; ^*∗∗*^*P* < 0.01, ^*∗*^*P* < 0.05 vs. the OVX group; ^Δ^*P* < 0.05 vs. the GBHD group. Mean ± SD, *n* = 3.

Compared with the sham group, the rats in the OVX group had significantly elevated serum levels of IL-6 and TNF-*α* (*P* < 0.01). Compared with the OVX group, IL-6 and TNF-*α* levels in the EV, GBHD, GBMD, and GBLD groups decreased significantly (*P* < 0.05), as shown in Figure 11.

#### 4.3.2. BMP-2, RUNX2, and ERK1/2 mRNA Expression

**Figure 12:**
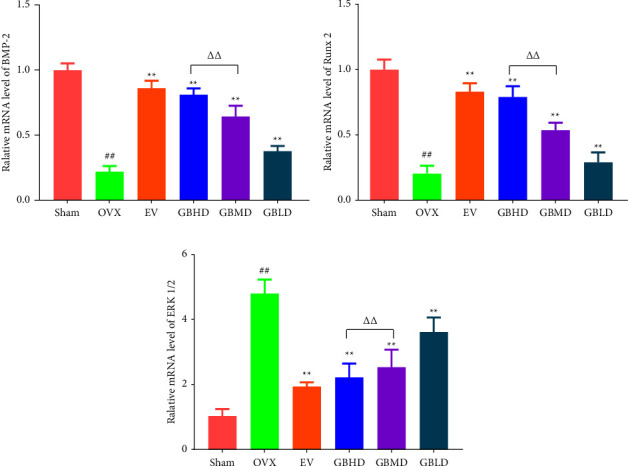
Relative expression of BMP-2, Runx2, and ERK1/2 mRNA. (a) Relative mRNA level of BMP-2; (b) Relative mRNA level of Runx2; (c) Relative mRNA level of ERK1/2. Mean ± SD, *n* = 3. ^##^*P* < 0.01 vs. the sham group; ^*∗∗*^*P* < 0.01, ^*∗*^*P* < 0.05 vs. the OVX group; ^ΔΔ^*P* < 0.01 vs. the GBHD group.

Compared with the sham group, the OVX group showed significantly reduced expression of BMP-2 mRNA (*P* < 0.01). Compared with the OVX group, the expression of BMP-2 mRNA was significantly increased in the EV, GBHD, GBMD, and GBLD groups (*P* < 0.01), as shown in Figure 12(a). Compared with the sham group, Runx2 mRNA expression was significantly decreased in the OVX group (*P* < 0.01). Compared with the OVX group, the expression of Runx2 mRNA was significantly increased in the EV, GBHD, GBMD, and GBLD groups (*P* < 0.05), as shown in Figure 12(b). Compared with the sham group, the expression of ERK1/2 mRNA was significantly increased in the OVX group (*P* < 0.01). Compared with the OVX group, the expression of ERK1/2 mRNA was significantly decreased in the EV, GBHD, GBMD, and GBLD groups (*P* < 0.01), as shown in Figure 12(c).

#### 4.3.3. BMP-2, ERK1/2, p-ERK1/2, and Runx2 Protein Expression

**Figure 13:**
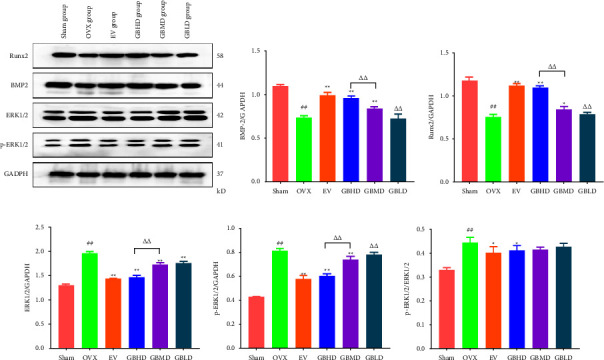
Expression of BMP-2, ERK1/2, p-ERK1/2, and Runx2 proteins. (a) Expression of BMP-2, Runx2, ERK1/2 and p-ERK1/2 protein was detected by western blotting; (b) BMP-2; (c) Runx2; (d) ERK1/2; (e) p-ERK1/2; (f) p-ERK1/2/ERK1/2. Mean ± SD. *n* = 3. ^##^*P* < 0.01 vs. the sham group; ^*∗∗*^*P* < 0.01, ^*∗*^*P* < 0.05 vs. the OVX group; ^ΔΔ^*P* < 0.01 vs. the GBHD group.

Compared with the sham group, BMP-2 expression was significantly lower in the OVX group (*P* < 0.01). Compared with the OVX group, BMP-2 was significantly higher in the EV, GBHD, and GBMD groups (*P* < 0.01), as shown in Figure 13(b). Compared with the sham group, the expression of Runx2 was significantly lower in the OVX group (*P* < 0.01). Compared with the OVX group, the expression of Runx2 was significantly higher in the EV, GBHD, and GBMD groups (*P* < 0.05), as shown in Figure 13(c). Compared with the sham group, the expression of ERK1/2 was significantly increased in the OVX group (*P* < 0.01). Compared with the OVX group, the content of ERK1/2 protein was significantly decreased in the EV, GBHD, GBMD, and GBLD groups (*P* < 0.01), as shown in Figure 13(d). Compared with the sham group, the expression of p-ERK1/2 expression was significantly increased in the OVX group (*P* < 0.01). Compared with the OVX group, the expression of p-ERK1/2 was significantly decreased in the EV, GBHD, and GBMD groups (*P* < 0.01), as shown in Figure 13(e). Compared with the sham group, the expression of p-ERK1/2 was significantly increased in the OVX group (*P* < 0.01). Compared with the OVX group, the expression of p-ERK1/2 was significantly decreased in the EV, GBHD, and GBHD groups (*P* < 0.05), as shown in Figure 13(f).

#### 4.3.4. Immunohistochemical Results

**Figure 14:**
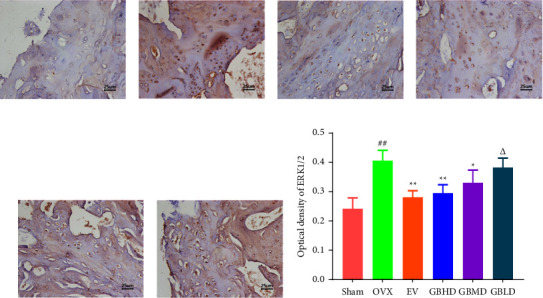
Immunohistochemical staining results of ERK1/2 in the rat femur. (a) Sham group; (b) OVX group; (c) EV group; (d) GBHD group; (e) GBMD group; (f) GBLD group; (g) optical density of ERK1/2. Mean ± SD, *n* = 3. Scale bar = 25 *μ*m. ^##^*P* < 0.01 vs. the sham group; ^*∗*^*P* < 0.05, ^*∗∗*^*P* < 0.01 vs. the OVX group; ^Δ^*P* < 0.05 vs. the GBHD group.

Compared with the sham group, the expression of ERK1/2 protein was significantly increased in the OVX group (*P* < 0.01). Compared with the OVX group, the content of ERK1/2 protein was significantly reduced in the EV, GBHD, and GBMD groups (*P* < 0.05), as shown in Figures 14(a)–14(g).

**Figure 15:**
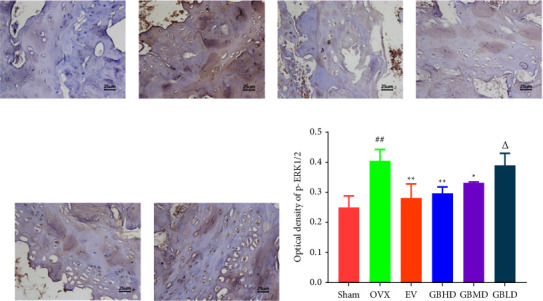
Immunohistochemical staining results of p-ERK1/2 in the rat femur. (a) Sham group; (b) OVX group; (c) EV group; (d) GBHD group; (e) GBMD group; (f) GBLD group. (g) Optical density of p-ERK1/2. Mean ± SD, *n* = 3. Scale bar = 25 *μ*m. ^##^*P* < 0.01 vs. the sham group; ^*∗*^*P* < 0.05, ^*∗∗*^*P* < 0.01 vs. the OVX group; ^Δ^*P* < 0.05 vs. the GBHD group.

Compared with the sham group, the levels of p-ERK1/2 protein were significantly increased in the OVX group (*P* < 0.01). Compared with the OVX group, p-ERK1/2 protein staining was significantly reduced in the EV, GBHD, and GBMD groups (*P*< 0.05), as shown in Figures 15(a)–15(g).

## 5. Discussion

**Figure 16:**
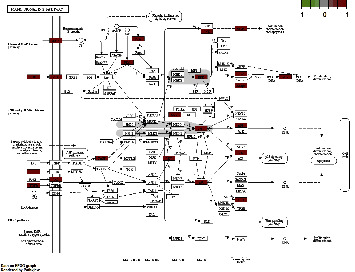
MAPK pathway.

Low bone mass and microstructural degeneration of bone tissue constitute the major lesion characteristics of OP, resulting in increased bone fragility and fracture risk [11]. The mechanism is intricately related to factors such as body hormone levels, genetic inheritance, lifestyle habits, underlying diseases, drug use, and exercise immobilization. This disease belongs to the category of “bone impotence” and “bone withering” in TCM. OP is manifested by kidney essence loss and loss of nutrition from bones. The primary symptoms include kidney deficiency and blood stasis, spleen, and stomach weakness. The treatment of OP is based on tonifying the kidneys, invigorating the spleen, and filling the essence [12]. GBZGG is composed of nine herbs: Radix Astragali, Angelicae Sinensis Radix, *Codonopsis pilosula*, Epimedii Folium, *Rehmannia glutinosa*, Cistanches Herba, Psoraleae Fructus, Cibotii Rhizoma, and Linderae Radix. We used network pharmacology to analyze the active components, target genes, and signaling pathways of GBZGG in treating OP. The results demonstrated that quercetin, luteolin, stigmasterol, angelicin, kaempferol, bakuchiol, 7-O-methylisomucronulatol, isorhamnetin, formononetin, and beta-sitosterol constitute the major components of GBZGG in treating OP. Quercetin, luteolin, beta-sitosterol, and kaempferol have been shown to exert important effects on bone metabolism, whereas the mechanism of action of other ingredients warrants further studies. Quercetin can maintain the balance of bone homeostasis through different signaling pathways, and Wong et al. [13] summarized the achievements of the application of quercetin in the field of bone metabolism research and found that quercetin can inhibit RANKL-mediated osteoclastogenesis, osteoblast apoptosis, oxidative stress, and the inflammatory response while promoting osteogenesis, angiogenesis, adipocyte apoptosis, and osteoclast apoptosis. Luteolin belongs to plant flavonoids, and a study demonstrated that luteolin inhibits the differentiation function of osteoclasts, increases bone mineral density, and reduces bone loss in OVX-induced mice, showing certain potential in preventing postmenopausal osteoporosis [14]. Beta-sitosterol is the most abundant phytosterol, and studies by Wang et al. [15] have stated that beta-sitosterol effectively prevents glucocorticoid-induced OP by protecting osteoblasts and inhibiting osteoclastogenesis. Kaempferol is a flavonoid with osteogenic activity, and studies have demonstrated that kaempferol reduces glucocorticoid-induced bone loss, enhances bone regeneration capacity at the fracture site, and exerts a positive effect on maintaining bone health [16]. The PPI network and cluster analysis revealed IL6, MAPK1, MAPK8, and MAPK14 as the core targets of GBZGG. The KEGG analysis of the consensus genes of TCM compound diseases demonstrated them to be involved in signaling pathways, including the MAPK signaling pathway. The MAPK signaling pathway family is intricately related to the balance of bone homeostasis and exerts dual regulatory effects on the process of bone formation and bone resorption via the MAPK/ERK signaling pathway, p38 signaling pathway, and JNK signaling pathway, as shown in Figure 16. Moreover, it is tightly related to the proliferation, differentiation, and apoptosis of cells [17]. The MAPK/ERK signaling pathway is one of the major signaling pathways of MAPK—an important pathway for major growth factors and cytokines to regulate cell proliferation and differentiation. It plays a crucial role in osteogenic differentiation as well as bone formation [18]. The MAPK signaling pathway can be activated by inflammatory factors, further increasing the expression of c-Fos and activating activated T cell nuclear factor 1 (NFATc1) to promote the differentiation and maturation of osteoclasts and accelerate bone resorption [19]. During OP, the activity of osteoclasts increases, accelerating the apoptosis of osteoblasts. Apoptotic cells stimulate the surrounding osteocytes and macrophages and secrete several factors such as RANKL, IL-6, and TNF-*α*. Inflammatory factors will promote the expression of ERK1/2 and its phosphorylation levels to accelerate the activation of osteoclasts and increase the RANKL/OPG ratio by activating ERK1/2 to promote the proliferation of osteoclasts [20]. Combined with the analysis results of network pharmacology, we speculated that GBZGG could affect bone metabolism via the MAPK/ERK signaling pathway via exerting the effect on the expression of inflammatory factors, as verified by animal experiments.

After establishing the OVX OP rat model, the serum levels of IL-6 and TNF-*α* were measured by ELISA. The levels of IL-6 and TNF-*α* were significantly increased in the OVX group, indicating altered levels of estrogen and activation of inflammatory factors following OVX modeling in rats. Considering that BMD is an essential indicator for evaluating bone health and the degree of osteoporosis, we detected the levels of BMD. The BMD of rats was significantly reduced after modeling to the extent of causing bone loss. Analysis of HE sections revealed that the structure of femoral tissue in the OVX group became thinner and sparsely arranged, the continuity was interrupted, the structure was incomplete, numerous bone blind ends appeared, the thickness of the trabecular wall was unequal, and osteoblast and osteoclast proliferation was observed around the bone, indicating that the bone immune microenvironment was overexpressed and the bone resorption activity was increased after castration in rats. Similarly, Weitzmann [21] reported that immune activation and bone loss initiated simultaneously during menopause, and decreased estrogen levels activated T cell hyperactivation, producing excessive IL-6 and TNF-*α*. The activation of bone resorption-related signaling pathways increased bone loss. Micro-CT, a scanning imaging technology, can be used to observe the detailed changes in bone structure as it more intuitively and accurately observes the microscopic changes in the bone following bone loss. It is an ideal method to observe the micromorphological changes in the bone [22]. To further observe the changes in bone microarchitecture, we applied micro-CT to scan the rat femur. The 2D scanning sections of different positions of femoral samples showed major bone loss, resulting in the disappearance of the bone cavity in the OVX group. The overall structure and quality changes in the cancellous bone in the femur were further evaluated after 3D pattern mapping and comparison of the content of bone in each group. Considering the overall structure of the skeleton, the cancellous bone will decay from a morphologically dense plate-like structure to a morphologically sparse rod-like structure during the development of the OP, thereby increasing the SMI. Simultaneously, since the rod-shaped structure of cancellous bone has a large gap, the Conn.D between bones decreases, and subsequently, the BV/TV used to describe the specific gravity of cancellous bone volume in this space also decreases, reflecting the degree of defect of the overall structure of cancellous bone at this site. After drug intervention, the levels of IL-6 and TNF-*α* in each group were reduced, and the BMD index was significantly improved, with the most significant effect in GBHD and EV groups. In addition, micro-CT results demonstrated that Tb.N and Tb.Th were significantly increased, SMI and Tb.Sp values were significantly decreased, and Conn.D and BV/TV values were significantly elevated in the femoral tissue of rats in the EV and GBHD groups. This suggests that GBZGG can act directly on the bones to enhance the quality of cancellous bone, repair defective bone, prevent the degeneration of bones, improve bone strength, and enhance the stability of the overall structure of cancellous bone in the femur.

To further clarify the positive regulation of GBZGG on bone metabolism, we conducted mechanistic studies. BMP-2, a member of the BMP family of growth factors, is primarily distributed in bone tissue and activates the production of bone formation-specific transcription factors by receiving extracellular stimulatory signals from osteoblasts, thus promoting bone formation [23], whereas Runx2 is a specific transcription factor for bone formation. BMP-2 can promote osteogenesis by activating ERK1/2 and increasing the expression of Runx2 by elevating its phosphorylation level [24]. RT-PCR and western blotting revealed that BMP-2 and Runx2 proteins and mRNA expression were significantly decreased in the OVX group, indicating the reduced osteogenic potential of rats following OVX treatment. After drug treatment, the expression of BMP-2, Runx2, and mRNA in the GBHD and EV groups was significantly increased, confirming that GBZGG promoted bone formation in rats. We next found that the ERK1/2 mRNA was highly expressed in the OVX group, whereas the GBHD and EV groups showed significantly decreased expression, indicating that GBZGG inhibited the expression of the *ERK1/2* gene. Combined with the detection results of serum IL-6 and TNF-*α* levels, the MAPK/ERK signaling pathway could be activated by inducing the expression of inflammatory factors, further improving the activity of osteoclasts to promote bone resorption. To assess this, we detected the expression of ERK1/2 and p-ERK1/2 proteins. The results showed that the expression of ERK1/2 and p-ERK1/2 proteins was significantly increased, whereas that of BMP-2 and Runx2 proteins was significantly decreased in the OVX group, indicating that ERK1/2 and p-ERK1/2 exacerbated bone resorption. The expression of ERK1/2 and p-ERK1/2 proteins was significantly decreased, whereas that of BMP-2 and Runx2 proteins was significantly increased after drug treatment, indicating that GBZGG inhibited the activation of ERK1/2 and p-ERK1/2 and reduced the occurrence of bone resorption. To further verify the expression of the ERK signaling pathway in OP, we detected the expression of ERK1/2 and p-ERK1/2 proteins in rat bone tissues using immunohistochemical staining. The results were consistent with those of western blotting. This proves that GBZGG promotes bone formation, not through the BMP-2-ERK1/2-Runx2 pathway for conduction but via the negative regulation of the MAPK/ERK signaling pathway. These results also explain from another perspective that the functional expression of the MAPK/ERK signaling pathway is affected by the overall environment of the body and is not the embodiment of a single function.

In all, combined with the above results, GBZGG improved the BMD levels, delayed the degree of bone loss and structural degeneration of the femoral bone in OVX model rats, and promoted bone formation by upregulating the expression of BMP-2, Runx2 protein, and mRNA. The negative regulation of the MAPK/ERK signaling pathway could be achieved by downregulating the expression of IL-6 and TNF-*α*, providing an experimental basis for the mechanism study of GBZGG in preventing and treating OP.

## Figures and Tables

**Table 1:** Primer sequence of target genes.

Gene	Sequence
GAPDH	5′-AATGGTGAAGGTCGGTGTG-3′(F)
	5′-AGGTCAATGAAGGGGTCGTTG-3′(R)
BMP-2	5′-ACCCGCTGTCTTCTAGTGTTG-3′(F)
	5′-AGCCTCAACTCAAACTCGCT-3′(R)
Runx2	5′-GTGTGATGGGTCCCTGCTTG-3′(F)
	5′-AACGCACAGCAGTACACAGA-3′(R)
ERK1/2	5′-AACCCAAACAAGCGCATCAC-3′(F)
	5′-CCACTGGTTCATCTGTCGGA-3′(R)

**Table 2:** Numerical changes in Tb.N, Tb.Th, and Tb.Sp (mean ± SD, *n* = 3).

Group	Tb.N (1/mm)	Tb.Th	Tb.Sp (mm)
Sham	4.231 ± 0.344	0.090 ± 0.003	0.223 ± 0.016
OVX	1.489 ± 0.058^##^	0.067 ± 0.002^##^	0.702 ± 0.036^##^
EV	3.048 ± 0.435^*∗∗*^	0.081 ± 0.001^*∗∗*^	0.363 ± 0.038^*∗∗*^
GBHD	2.925 ± 0.254^*∗∗*^	0.079 ± 0.001^*∗∗*^	0.352 ± 0.030^*∗∗*^
GBMD	2.341 ± 0.105^*∗∗*Δ^	0.075 ± 0.001^*∗∗*Δ^	0.545 ± 0.014^*∗∗*ΔΔ^
GBLD	1.840 ± 0.073^△△^	0.071 ± 0.001^*∗*ΔΔ^	0.442 ± 0.041^*∗∗*ΔΔ^

Note. Compared with the sham group, ^##^*P* < 0.01; compared with the OVX group, ^*∗∗*^*P* < 0.01, ^*∗*^*P* < 0.05; compared with the GBHD group, ^ΔΔ^*P* < 0.01, ^Δ^*P* < 0.05.

**Table 3:** Numerical changes in Conn.D, SMI, and BV/TV (mean ± SD, *n* = 3).

Group	Conn.D (1/mm^3^)	SMI	BV/TV (%)
Sham	110.14 ± 5.94	0.609 ± 0.154	0.321 ± 0.011
OVX	26.58 ± 1.99^##^	2.153 ± 0.140^##^	0.096 ± 0.002^##^
EV	70.93 ± 7.30^*∗∗*^	1.305 ± 0.189^*∗∗*^	0.211 ± 0.004^*∗∗*^
GBHD	71.69 ± 3.44^*∗∗*^	1.387 ± 0.228^*∗∗*^	0.206 ± 0.007^*∗∗*^
GBMD	45.14 ± 4.94^*∗*ΔΔ^	1.692 ± 0.063^*∗∗*Δ^	0.157 ± 0.006^*∗∗*ΔΔ^
GBLD	32.17 ± 3.24^△△^	1.916 ± 0.109^△△^	0.120 ± 0.004^*∗∗*ΔΔ^

*Note.* Compared with the sham group, ^##^*P* < 0.01; compared with the OVX group, ^*∗∗*^*P* < 0.01, ^*∗*^*P* < 0.05; compared with the GBHD group, ^ΔΔ^*P* < 0.01, ^Δ^*P* < 0.05.

**Table 4:** MCODE cluster analysis detail sheet.

Cluster	Network	Nodes	Edges	Node IDs
1	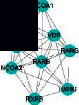	9	32	NCOA3, NCOA2, RXRB, RXRG, VDR, RXRA, RARB, RARG, NCOA1

2	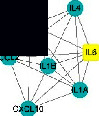	7	20	IL1B, IL1A, IL4, CXCL8, CCL2, CXCL10, IL6

3	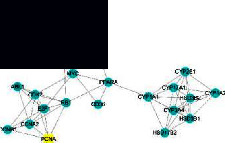	22	59	HSD17B2, FOS, HSD3B1, HSD3B2, CCNB1, CYP1A1, PCNA, CYP17A1, PPARG, CYP3A4, RELA, CD36, E2F1, PPARA, CYP2E1, MYC, CCNA2, RB1, CDK2, ABL1, NR3C1, CYP1A2

4	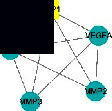	5	9	MMP3, MMP2, MMP1, VEGFA, MMP9

5	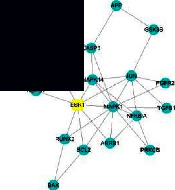	19	37	APP, GSK3B, CASP3, ESR1, FAS, RUNX2, PARP1, JUN, CASP7, HSPB1, ARRB1, MAPK14, TGFB1, BAX, FGFR2, NFKBIA, PRKCB, MAPK1, BCL2

6	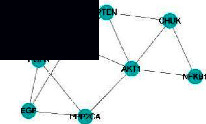	9	16	CHUK, IGFBP3, EGF, CAV1, PTEN, PPP2CA, EGFR, NFKB1, AKT1

7	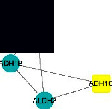	4	5	ADH1B, ADH1C, ADH1A, ALDH2

8	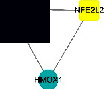	3	3	POR, NFE2L2, HMOX1

9	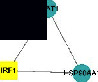	3	3	IRF1, STAT1, HSP90AA1

10	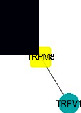	3	3	TRPA1, TRPV1, TRPM8

11	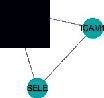	3	3	SELE, ICAM1, VCAM1

12	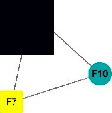	3	3	F7, F3, F10

13	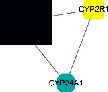	3	3	CYP27B1, CYP2R1, CYP24A1

14	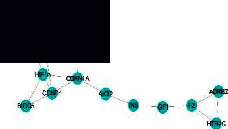	15	20	IGF1, ADRB2, INS, AKT2, CDKN1A, MAPK8, BIRC5, NOS3, AR, KDR, HIF1A, PRKACA, CCND1, F2, HTR2C

## Data Availability

The datasets generated during and/or analyzed during the current study are available from the corresponding author on reasonable request.

## Supplementary Materials

Supplementary Materials Supplementary 1. Compounds and related targets genes of GuBen-ZengGu granules. Supplementary 2. The targets of osteoporosis. Supplementary 3. The interacting genes between osteoporosis and GuBen-ZengGu granules. Supplementary 4. The GBZGG-ingredient-target-OP interaction network. Supplementary 5. The key ingredients. Supplementary 6. Topological analysis results. Supplementary 7. MCODE analysis results. Supplementary 8. KEGG enrichment analysis results. Supplementary 9. The component-disease-pathway-target interaction network.
